# Patient-Centric Care for Parkinson's Disease: From Hospital to the Community

**DOI:** 10.3389/fneur.2020.00502

**Published:** 2020-06-09

**Authors:** Yin Minn Aye, Sylvia Liew, Shermyn Xiumin Neo, Wei Li, Hwee-Lan Ng, Shu-Ting Chua, Wen-Tao Zhou, Wing-Lok Au, Eng-King Tan, Kay-Yaw Tay, Louis Chew-Seng Tan, Zheyu Xu

**Affiliations:** ^1^Department of Neurology, National Neuroscience Institute, Singapore, Singapore; ^2^Parkinson Disease and Movement Disorders Centre, Parkinson Foundation Centre of Excellence, National Neuroscience Institute, Singapore, Singapore; ^3^Department of Neurology, Yangon General Hospital, Yangon, Myanmar; ^4^Parkinson Society Singapore, Singapore, Singapore

**Keywords:** Parkinson's disease, multidisciplinary approach, community care, telemedicine, Singapore model of care

## Abstract

Parkinson's disease (PD) is a chronic neurodegenerative disease with complex motor and non-motor symptoms often leading to significant caregiver burden. An integrated, multidisciplinary care setup involving different healthcare professionals is the mainstay in the holistic management of PD. Many challenges in delivering multidisciplinary team (MDT) care exist, such as insufficient expertise among different healthcare professionals, poor interdisciplinary collaboration, and communication. The need to attend different clinics, incurring additional traveling and waiting time for allied health therapies can also make MDT care more burdensome. By shifting MDT care to local community settings and into patients' homes, patient-centered care can be achieved. In Singapore, the National Neuroscience Institute created the Community Care Partners Programme in 2007 to bring the allied MDT team to the community and nurse-led Integrated Community Care Programme for Parkinson's Disease in 2012 to provide care in community and at patient's home. However, attaining MDT care in the community setting is difficult to achieve where there is a shortage of PD-trained professionals. As such, interdisciplinary and transdisciplinary management would be other best practice options to deliver patient-centric care in PD. Telemedicine could be another viable option to bring the MDT closer to the patient.

## Introduction

In Parkinson's disease (PD), multidisciplinary care is widely practiced in hospital settings to manage the multiple symptomatology of this complex disorder. However, the provision of multidisciplinary care in the hospital setting can be challenging in resource-tight healthcare systems, resulting in delays and reactive rather than proactive care. One of the ways to provide patient-centered care is to bring care directly to the patients in the local community setting or straight into the home environment.

Patient-centric care has been defined as “providing care that is respectful of, and responsive to, individual patient's needs and preferences” (1). A recent review of delivering patient-centered care in PD highlighted the importance of patient education and multidisciplinary care, along with the use of patient-centered outcomes to better capture the patient experience and improve the delivery of individualized therapy (1).

This review aims to present the current evidence and knowledge gaps in multidisciplinary care and provision of community care in the PD and review the evidence for telemedicine use. We showcase how our model of community care in PD that includes delivery of allied health interventions in the community, provision of physiotherapy (PT) through group exercises with a non-governmental organization (NGO), and home care services was implemented to supplement specialist, hospital-based PD care within a public health setting in Singapore. We also highlight the recent applications of telemedicine in the field that could assist in improving delivery care models in PD.

## Role and Evidence for Multidisciplinary Care in PD

PD is a chronic, degenerative neurological disorder with a complex and heterogeneous phenotype. As disease progresses, patients show motor symptoms and non-motor symptoms that do not always respond to pharmacological therapies. Thus, an integrated, multidisciplinary approach that assembles different healthcare professionals is necessary to achieve holistic care (2). There has been an increasing drive toward providing patient-centered rather than physician-centered care (3).

In recognition that collaboration may improve patient care, healthcare professionals are increasingly required to work together to share expertise, knowledge, and skills. In transdisciplinary care, team members work jointly using a shared conceptual framework that draws together concepts, theories, and approaches from multiple disciplines in order to define, address, and resolve complex real-world problems, whereas in the interdisciplinary approach, they are working together but from individual disciplinary perspectives to address a common problem (4). Multidisciplinary care, on the other hand, is working in parallel or sequentially from disciplinary-specific frames to address common problems (4). The preferred care model will vary depending on the patient and disease profile and the local healthcare infrastructure. Multidisciplinary care is currently the most established model of care for PD.

Components of a PD multidisciplinary team (MDT) vary significantly according to the local practices and the availability of allied healthcare professionals. The MDT team is typically tertiary hospital-based to be able to assemble a diverse range of allied healthcare professionals (5). More ambitious models involving medical professionals from other less traditional fields of vascular medicine, urology, gastroenterology, geriatric medicine, and palliative care have been proposed (6).

Because of the heterogeneity of interventions and outcomes used, the evidence on multidisciplinary rehabilitation in PD remains limited and fail to reveal consistent long-term benefits particularly for objectively motor outcomes (2, 7). In contrast, subjective outcome measures have generally yielded positive results, but the placebo effect of dedicated allied health interventions within a clinical trial setting cannot be ignored. However, it can also be argued that MDT interventions are not amenable to testing in experimental settings unlike other single interventions in medical research. van der Marck et al. studied the effects of MDT care in Canada as a single-blind randomized controlled trial (RCT) and showed improvements in quality of life, motor function, depression, and social function scores after 8 months (8). However, van der Marck et al. later published the landmark IMPACT study in the Netherlands comparing community with integrated care interventions with those that gave usual care: small benefits were achieved in disability scores and quality of life, which had disappeared when corrected for baseline disease severity (9). Similarly, a UK study showed that although MDT intervention improved psychological well-being, this required ongoing care to maintain long-term benefits. Ferrazoli et al. in Italy studied the effects of a 4-weeks intensive hospital-based therapy: at 3 months, only PDQ-39 showed improvement, but not other objective motor outcome measures (10).

Potential limitations to the implementation of effective MDT intervention are distance, insufficient expertise among the different health professionals, poor interdisciplinary collaboration, and high costs. Furthermore, MDT care often requires patients to traverse between different clinic settings, incurring additional waiting and traveling time for routine follow-up appointments or allied health therapies.

There has been an increasing push to deliver more patient-centered care: two approaches that have been explored in recent years include bringing models of care to the community and providing care via telemedicine (6).

## Community Care: Shifting Care to the Community

Even at the early disease, PD patients report physical limitations and early rehabilitation is vital in maintaining function (11). However, in one large RCT, personalized home PT interventions did not reduce fall rates (12). Subgroup analyses showed that only patients with milder disease benefitted.

Community-based group balance exercises classes in patients with early PD can lead to self-reported improvements in balance control while also meeting their social and emotional needs (11), which may improve adherence. In a UK 2-arm RCT with blinded assessment, a minimally supported community exercise intervention delivering twice weekly 30-min aerobic and 30-min resistance training over 6 months, both gait speed and MDS-UPDRS Part III scores improved (13).

Additionally, community-based care may allow for care to be provided at lower cost. Using a Discrete Event Simulation model applied to the National Health Service in the UK, shifting patients with PD from hospital to community services allowed reductions in hospital visits by a quarter and reduction in hospital manpower by one-third. Hospital-based treatment costs were estimated to decrease by 26%, leading to 10% overall savings in the total cost of treating PD patients (14).

One of the most successful and established model of regional care is the ParkinsonNet model in the Netherlands (15), where medical and allied health interventions are delivered within integrated regional community networks dispersed throughout the country by PD-specific therapists with specialized training who manage high caseloads (16). Better quality of care, fewer PD-related complications, lower mortality risk, and lower total healthcare costs were achieved compared to usual care (17, 18), and the use of specialized occupational therapy delivered in the community setting resulted in an improvement in self-perceived daily functioning (19).

While ParkinsonNet has been introduced to other European countries (20), its applicability has limited generalization across other healthcare systems. Singapore has been instead working toward an interdisciplinary approach for community care that allows the healthcare professionals to adopt some roles of the other professionals.

## Overview of the Singapore Model of Care

PD is the second most common neurodegenerative disease in Singapore with an estimated 6,000 patients. The National Neuroscience Institute (NNI), which is one of the designated Parkinson centers of Excellence (PCOE), looks after approximately two-thirds of the patients in the public health setting in specialized tertiary movement disorders clinics staffed by movement disorder neurologists and supported by specialist allied health professionals who deliver PD-specific care. Within our catchment area, we have an approximate ratio of movement disorder neurologists to PD patients of 1:500. However, despite Singapore's small geographic size (721.5 km^2^), patients in advanced disease remain dependent on caregivers or on private ambulance services to access in-hospital care. Allied health reviews are scheduled with their twice-yearly medical appointments where possible, but this is logistically challenging to achieve when multiple MDT input is required. A substantial proportion of our patients thus chose to forgo allied health visits.

As medical costs are still partially borne by patients who are means-tested, with each patient paying according to his/her ability, affordability of care is important. In 2007, we brought the allied MDT team to the community by establishing the Community Care Partners Programme (CCPP), which was funded by an initial grant from the Parkinson Foundation but subsequently with costs borne by the patient as a sustainable model of care. This limited, low-cost service delivery model was based on the ParkinsonNet model, training nurses and allied health professionals, particularly physiotherapists, occupational therapists, and speech therapists, in PD-specific care through our annual education program. This half-day program is taught by the medical, nursing, and allied health professionals based at the PCOE, consisting of a short series of lectures and innovative small group teaching model in the form of practical stations to teach PD-specific skills, such as PT interventions to manage freezing of gait, walking stability and balance, bed transfers of patients, and mobility of patients from a vehicle. This interdisciplinary education class allows our allied health partners to be upskilled in the care of PD patients.

In our model, patients with complex rehabilitation issues will continue to have their allied health reviews at the tertiary hospital. For patients with less complex issues, they are referred to allied health partners in the community, chosen for their proximity to the patient's home to allow greater access and encourage compliance to allied health interventions. The majority of our program is focused on delivering PT or speech therapy interventions, which can be carried out in individual or group settings. Delivery of allied health interventions in group settings has been one of the ways to maintain affordability of care and increase patient interactions.

The NNI oversees the training competency of our community partners in the form of yearly hands-on educational seminars conducted by our hospital-based MDT team. Our community partners are also invited to attend, at reduced costs, a symposium organized biennially where the most up-to-date advances in PD care are presented by an international and local faculty. Community care partners who have met our standards of care are acknowledged during our biennial symposiums and are invited to join our CCPP. In the past 13 years, we have established a network of 27 community partners with more than 400 allied healthcare providers trained to deliver specialist PD care in the community, thus freeing up hospital-based resources for patients requiring the most complex care.

Since 2014, we have also established a working collaboration with the Parkinson Society Singapore, an NGO, to support PD patients and their carers through the provision of allied health interventions as well as other social or wellness activities. Exercise programs include group PT exercises, non-contact boxing, and Tai chi classes. The organization has also collaborated with a large community care partner, St Luke's Eldercare, to train physiotherapists from 16 of their Day Rehabilitation Centers to conduct PT group classes. These centers allow greater access to specialized rehabilitation facilities in the community to ensure high standards of care.

Another aspect of providing community care is through the Integrated Community Care Programme for Parkinson's Disease (ICCP). This is a nurse-led service that was established in 2012 with funding from the Ministry of Health and the Tote Board Community Health Care Fund, and specifically targeted patients with more severe motor impairment or those without caregivers. Patients were visited by a specialist PD nurse who could refer them onwards to relevant community services if required. A training component was subsequently added to this model of care that allowed a larger network of 19 nurses from various homecare service providers from non-profit organizations to provide homecare. By 2018, 295 patients benefitted from this program.

As such, we are currently in the process of expanding the ICCP programme by (1) establishing a 3-months program of interprofessional training to community care partners; (2) providing patient-centered care by delivering care through community care partners, including joint consultations at the patient's home with movement disorders neurologists and the provision of care delivered by telemedicine; and (3) providing ongoing training in the form of monthly interprofessional learning, yearly access to our training programs, and biennial access to our symposium.

## Telehealth in PD

Telemedicine refers to the remote delivery of healthcare using telecommunications technology (21). Telemedicine for PD typically involves the use of videoconferencing platforms, as well as the remote use of devices, such as motion sensors to measure symptoms outside clinical settings (22). However, the spectrum of telemedicine is wide and includes telephone consults asynchronous communications via emails or text messaging (AMA). The progressive nature of PD makes it an ideal chronic condition to be managed via telemedicine, as patients and their caregivers often find it difficult to access care (23).

Telemedicine's feasibility has been established, particularly in large countries where greater time savings can be achieved. In a large RCT carried out in the US, telemedicine as an adjunct to usual care was shown to be feasible, with care standards comparable to in-person care (24). Another study was able to achieve an average of 3 h of time savings and reduce travel distance of up to 100 miles (25). However, patients generally preferred telemedicine to be combined with in-person visits rather than standalone telemedicine visits (26), and video quality is generally inadequate to allow accurate motor scoring (27). The UPDRS can be performed remotely with the exception of rigidity and postural instability testing (28).

The uptake of telemedicine in neurology has been slow. However, at the time of writing, we are experiencing a global pandemic of a novel coronavirus, Covid-19. This unprecedented event accelerated the adoption of tele-neurology, with loosening of policy and administrative restrictions (29). Implementation requires changes in legislation, reimbursement policies, and hospital workflows, including scheduling, billing, and prescribing practices (29). In addition, special attention needs to be paid to cybersecurity to safeguard patients' privacy. Many organizations, including the American Academy of Neurology and the International Parkinson and Movement Disorder Society, have issued guidelines on telemedicine use (30, 31).

At our institution, telephone and asynchronous consults via emails and text messaging have been in use for the last 5 years, to allow access to our specialist nurses. Our nurses have been empowered to troubleshoot disease-related complaints and adjust medications. This service is particularly reassuring for our deep brain stimulation patients who are in the initial programming phase and may help reduce clinic or emergency room visits.

We initiated our video consultation service in March 2019, choosing our primary service platform due to its reduced requirement for bandwidth without compromising on audiovisual performance. Additionally, the chosen service platform company already had a contractual service agreement in place to comply with our country's personal data protection act. Our operations team has worked closely with the primary service platform to improve the experience of video consultation, including requiring password authentication for meetings, having a waiting room feature to allow identity verification, and limiting content sharing to the provider.

Patient selection is vital with the service limited to those with stable neurological disorders, with at least one in-person consultation in the past year, who are able to cooperate or have a caregiver to assist. Written consent is obtained prior to the video consultation. Staff training in the form of hands-on training by IT support staff and e-learning modules are provided.

Telerehabilitation has been explored as a means of delivering allied health interventions remotely to improve balance (32) and deliver a web-based aerobic-based exercise program (33). Telerehabilitation has been successfully used to deliver speech therapy interventions, which are typically very time-intensive (34). At our center, we are in the process of implementing telerehabilitation for speech therapy and PT interventions. In the future, there are plans to collaborate with our homecare nursing teams to use telemedicine to optimize the care of homebound patients.

While there is great potential for telemedicine in the care of PD patients, it must be borne in mind that published studies in telemedicine have largely originated from developed countries with good infrastructure. Physicians generally rated telemedicine less favorably than patients, with technical problems in the software and inability to perform a detailed motor examination as the main caveats hindering more widespread adaptation of the technology (35).

Although there has been an explosion of studies exploring the use of sensors and smartphone applications that allow for data collection outside clinic settings to augment patient care, significant challenges persist in the form of non-compatible technology platforms and limited real-world clinical applications; hence, the Movement Disorders Society Task Force on Technology was convened in 2016 to set the direction of future research in this rapidly developing field (22).

## Clinical Indicators

Clinical indicators may be defined as measures that assess a particular healthcare process or outcome; quantitative measures to monitor and evaluate the quality of important management, clinical, and support functions that affect patient outcomes; or measurement tools, screens, or flags that are used as guides to monitor, evaluate, and improve the quality of patient care (36). Two PD groups have proposed their sets of quality measurement indicators in hospital-based settings, as summarized in Table 1 (37, 38).

**Table 1:** Quality and outcome measures for Parkinson's disease (PD).

**AAN Set (2015) (37)**	**International Consortium Set (2017) (38)**
1. Annual PD diagnosis review 2. Avoidance of dopamine-blocking medications 3. Psychiatric symptoms assessment 4. Cognitive impairment or dysfunction assessment 5. Querying about symptoms of autonomic dysfunction 6. Querying about sleep disturbance 7. Falls outcome 8. PD rehabilitative therapy options 9. Counseling about regular exercise regimen 10. Querying about PD medication-related motor complications 11. Advanced care planning	1. Cognitive and psychiatric symptoms/functioning 2. Non-motor functioning 3. Motor functioning 4. Additional health outcomes a. Ability to work b. Hospital admissions c. PD-related health status d. Falls

Many of these measurements may also be applied to telemedicine consults. More efforts are needed to derive a specific set of clinical indicators for PD community care. Meanwhile, we propose that emphasis should be given to falls outcomes, provision of rehabilitation therapy options, counseling about regular exercise, and assessment of quality of life health status.

## Conclusions

We present the Singapore model of community care that has been sustainable over 14 years. Although there is a low level of evidence for MDT interventions in PD, largely due to the heterogeneous nature of the interventions and the need for personalization that makes it difficult to study trial settings, MDT care in PD is widely practiced internationally and recommended in several guidelines (39). However, the formation of a MDT is uncommon in the community settings where there is a shortage of professionals with PD-specific expertise. Interdisciplinary and transdisciplinary management would be another feasible method to tap on the lean manpower where the professionals are cross trained to deliver community care (29). In addition, patient-centric care is less fragmented when all professionals' boundaries are blurred unlike in the traditional multidisciplinary approach. New care models based on recent technological developments that combine remote monitoring and self-monitoring may allow for a decrease in hospital care and allow truly patient-centric care to be achieved (40).

## Author Contributions

YA, SL, and SN: conception and design, and writing of the first draft of the manuscript. WL, H-LN, S-TC, W-TZ, W-LA, E-KT, and K-YT: review and critique of the manuscript. LT and ZX: conception and design, writing, review, and critique of the manuscript.

## Conflict of Interest

The authors declare that the research was conducted in the absence of any commercial or financial relationships that could be construed as a potential conflict of interest.
